# Where Did FinTechs Come From, and Where Do They Go? The Transformation of the Financial Industry in Germany After Digitalization

**DOI:** 10.3389/frai.2020.00008

**Published:** 2020-03-10

**Authors:** Barbara Brandl, Lars Hornuf

**Affiliations:** ^1^Faculty of Social Science, Institute of Sociology, Goethe University Frankfurt, Frankfurt, Germany; ^2^Faculty of Business Studies and Economics, University of Bremen, Bremen, Germany; ^3^Max Planck Institute for Innovation and Competition, Munich, Germany; ^4^Center of Finance, University of Regensburg, Regensburg, Germany; ^5^CESifo, Faculty of Economics, University of Munich (LMU), Munich, Germany

**Keywords:** application programming interface, API, crowdfunding, financial technology, FinTech, financial industry, IT infrastructure, robo-advice, L86, M13, O16, O32

## Abstract

The digitalization of financial services opened a window for new players in the financial industry. These start-ups take on tasks and functions previously reserved for banks, such as financing, asset management, and payments. In this article, we trace the transformation of the industry after digitalization. By using data on FinTech formations in Germany, we provide first evidence that entrepreneurial dynamics in the FinTech sector are not so much driven by technology as by the educational and business background of the founders. Furthermore, we investigate the reactions of traditional banks to the emergence of these start-ups. In contrast with other emerging industries such as biotechnology, a network analysis shows that FinTechs have mostly engaged in strategic partnerships and only a few banks have acquired or obtained a financial interest in a FinTech. We explain the restraint of traditional banks to fully endorse the new possibilities of digitalized financial services with the characteristics of the technology itself and with the postponed fundamental decisions of banks to modernize their IT infrastructure.

## Introduction

A key question in the economics of innovation literature is how industries change as they absorb technological breakthroughs. Are the existing companies able to incorporate the new technologies in their business routines? Does the emergence of new technologies open a window for new types of companies and thus reshape the structure of the industry? How does this change process affect the central institutions and organizations? Mapping the genesis of a new or transformed industry is a challenge because it is a multi-layered process that is both shaped and determined by the emerging technologies and their interplay with the existing institutions in the industry. Therefore, most research works backward from contemporary cases to develop a story about how institutions and organizations were purposefully created and rationally chosen to meet the upcoming challenges (Powell et al., 2012). In this article, we analyze the current transformation process of the financial industry. Who are the new players? When and where did they emerge? How did the type of technology shape this process? How do incumbents react to the challengers?

Digitalization has significantly challenged many traditional industries. This is especially true for the communication industry, the entertainment and media sector, and, more recently, the financial industry. The innovation that has the potential to turn the financial industry upside down is the digitalization of banking business segments such as financing, asset management, and payments. In the past, banks have been able to integrate digital financial innovations, such as Internet banking, and have established new digital technological infrastructures, such as SWIFT or TARGET2-Securities. However, most of the financial innovations were absorbed in the digital back end of banks where customers only indirectly benefited from them. The digitalization of front-end services has created opportunities for new companies. The emerging players in the financial sector are called FinTechs, an acronym for start-ups that commercialize technological financial innovations. Although these new start-ups are a heterogeneous group with diverse interests and business plans, they all have one thing in common: they take on tasks and functions that were traditionally reserved for banks (Puschmann, 2017).

FinTechs can roughly be grouped into four categories: financing (e.g., crowdfunding, crowdlending, crowdinvesting), asset management (e.g., robo advice, social trading, factoring), payments (e.g., crypto currencies, alternative payment systems), and other (e.g., search engines, infrastructure providers)^1^. In the past decade, the number of FinTech start-up formations and market volume in all four segments were steadily growing (Dorfleitner et al., 2017). In 2016, the total volume of all FinTechs in the segments of financing and asset management active in the German market was 7.9 billion EUR (Dorfleitner et al., 2019). The total transaction volume processed through FinTechs in the payment segment was estimated to be 17 billion EUR in 2015 (Dorfleitner et al., 2017).

The traditional players in the financial sector, however, have only reluctantly participated in these new technological possibilities especially in the areas of financing and asset management. This is only partly true for the payment segment. The two main initiatives to digitalize payment systems are the online payment system paydirekt, which is available to customers of around 1,400 German banks and savings banks^2^, and the instant payment system RT1, which was launched in January 2017 by 40 European Bank and provides a real-time processing facility for pan-European payments. However, traditional players in the financial sector have only reluctantly participated in the new technological possibilities of digitalized financial services and their market penetration is still small as compared, for example, to the mobile payment incumbent PayPal. Although recent years have witnessed some acquisitions of FinTechs by banks, most FinTech start-ups are not yet controlled by banks.

Despite the rapidly changing environment in the financial industry, almost no studies have investigated the FinTech–bank relationship and how the emergence of FinTechs affects the traditional banking sector. A notable exception is the study of Cumming and Schwienbacher (2018), who investigate the pattern of venture capital investments in FinTechs around the world. They find that venture capital investments in FinTechs can be attributed to differences in the enforcement of financial regulation among start-ups and banks after the financial crisis. Haddad and Hornuf (2019) evidence that countries witness more FinTech start-up formations when the economy is well-developed, venture capital is readily available, and people have more mobile telephone subscriptions. The available labor force and the number of secure Internet servers increase the number of FinTech start-ups in a country as well. Puschmann (2017) defines the term FinTech and presents a categorization of the phenomenon. More recently, Hornuf et al. (2018) have investigated the factors that drive banks to form alliances with FinTechs in Canada, France, Germany, and the United Kingdom. They find that banks are significantly more likely to form alliances with FinTechs when they pursue a well-defined digital strategy and/or employ a Chief Digital Officer. Furthermore, they evidence that markets react more strongly if digital banks rather than traditional banks announce a bank-fintech alliance.

However, to the best of our knowledge, no study has examined the genesis of the transformation of the industry and its interplay with the dominate players in the financial sector. To close this gap in the literature, we chose an explorative approach to map the emergence of FinTechs in the German financial industry. To gain a deeper understanding of these processes, we combine insights from transaction cost theory and concepts of economic sociology. Empirically, we trace this process by using data on the FinTech founders and their professional biographies. Furthermore, we collect data on investments and strategic cooperations of banks with FinTechs in Germany. We conduct a simple network analysis based on this dataset. Theoretically, we base our analysis on insights from transaction cost theory (Coase, 1937; Williamson, 1981) and its further development by organizational theorists, who provide a specific focus on technology development (Teece, 1986, 1998).

This paper proceeds as follows: section Innovation in the Financial Industry provides an overview of the development and the current state of digitalization in the financial sector. We also address the difficulties and the potential of digitalization in the financial service industry. Section Methods and Data presents our data and method. In section Results, we present our findings and argue that the limitation of banks to fully integrate FinTechs can be explained by the characteristics of the technology and postponed fundamental decisions of banks regarding their IT infrastructure. In section Conclusion, we conclude that the future of digital financial innovations will not be decided by technological superiority but by institutional factors. Thus, the future diffusion of digital financial innovations rests on a coordination problem, the solution of which depends on the establishment of novel, effective institutions and organizations.

## Innovation in the Financial Industry

Merton (1992) identifies four core functions of financial services that innovation needs to address: (1) the moving of funds across time and space (e.g., saving accounts, credit cards), (2) the pooling of funds (e.g., stock markets), (3) managing risk (e.g., derivative products), and (4) extracting information to support decision making and to address asymmetric information problems (e.g., markets for products that deal with default probabilities such as swaps). Given these specific requirements, innovations in finance differ in many respects from the innovations in other fields. Because of the specific features of innovations in the financial industry, financial innovations were rarely the subject of traditional innovation studies and their inquiries. A noteworthy exemption is Awrey (2013), who argues that innovation in the financial sector can only insufficiently be understood by the neoclassical concept of innovation, which describes innovation as a rational answer to market frictions. Instead, he suggests a theoretical perspective in which law in the form of public regulation and private contractual agreements is regarded as a catalyst for innovation in the financial sector. Lerner and Tufano (2011) suggest that innovations in finance contain dynamics that differ from innovation processes in other fields, because the technology behind financial innovation is rather trivial. Digitalization enabled the definition of atomic small business-to-business and business-to-consumer services, which has changed the structural conditions of the financial industry and the possibilities for innovation. As a result, technological innovations are no longer excluded from the financial sector but deeply interwoven in the creation of new firms and financial products.

Significant technological innovations in the financial industry began in the 1960s with the installation of ATM machines and continued with the computerizing of core banking operations (Millo et al., 2005). Today, digitalization has enabled start-ups to extract profitable parts of banking operations in market segments that were previously not often catered to by banks. For example, crowdfunding is the practice in which entrepreneurs raise capital for a project or product from the larger public, often without a securities prospectus. Crowdfunding can be either reward based (the investor pre-purchases a product or service) or equity based (investors pool money to support a project or company). Two functions of financial innovation—that is, moving of funds in time and the pooling of funds from non-sophisticated investors—became possible through new technologies: online platforms that provide an infrastructure to connect individuals who are willing to invest in artists, start-ups, or non-governmental organizations that want to raise money for their projects. The digitization of financial services, however, not only implies a new way of providing financial services but also questions the traditional relationships between lenders and borrowers and between entrepreneurs and customers and thereby challenges the dominant position of banks.

Although the digitalization of *financial services* has so far been portrayed as novel, it would be wrong to consider the digitalization of the *financial industry* a recent phenomenon. The expenditures of the financial sector for IT devices and services have traditionally been rather high. By 1979, the financial industry had already dedicated 32% of all expenses to IT, which was the highest share of all sectors, a number that even increased to 38% in 1992 (Scott et al., 2017). The high share of IT expenses can be explained by the financial sector being the first industry to employ computers on a large scale in its work processes. The first wave of adaptation to the early telecommunication and information technologies had already begun in the late 1950s and peaked in the 1980s. Franke (1987) states that in 1980, half of banks' fixed capital expenditure was for computers or in some form computer related. As a result, the digital architecture of the financial system as well as the internal business routines of banks date back to that time.

This early adoption of computers by the financial industry was possible through Common Business-Oriented Language (COBOL), a problem-oriented programming language that was developed in 1959 as one of the first languages to program business applications. While the early programming languages were predominately used for scientific purposes, COBOL is a hardware-independent software that has the capacity to access and manipulate masses of data (Beyer, 2012). Although in the meantime other more manageable and speedier programming languages such as Java or Python have become available, the clear majority of software applications of banks and credit card companies are still based on COBOL and mainframes.

The outdated IT infrastructure is at least partly responsible for the difficulties banks are facing today in the digitalization of financial services. Although the current infrastructure is highly resilient and robust, it is also very costly to maintain and update. COBOL performs well in the traditional core activities of banks, such as the daily settlement of payments, but is monstrously complex and not well-suited to integrate fast and flexible applications. While the growth rates of IT expenses in the financial industry are still above average today, it appears that traditional banks must invest much more to replace the existing IT infrastructure.

## Methods and Data

To examine where FinTechs come from and how the financial industry has changed since it began absorbing digital innovations, we use a mixed-methods approach. To learn more about why FinTech have emerged, we first investigate the educational and professional background of the founders. If FinTech is a technology-driven activity or, in line with Awrey (2013), is a result of legal arbitrage opportunities, this should to some extent be reflected in the founder backgrounds. To describe the current state of collaboration and consolidation, we then conduct a social network analysis of the cooperation and investment activities of FinTechs and the financial industry.

### Method and Data

To investigate how FinTechs interact with banks, we conduct a network analysis, which enables us to gain a better understanding of the current market structure through visualization techniques (Powell and Grodal, 2005; Powell et al., 2005; Scott and Carrington, 2011). Our network of banks and FinTechs in the German market is represented as a graph constructed of *nodes* (companies) and *links* (type of connection between companies). In particular, we differentiate between three types of nodes—banks, FinTechs, and FinTech banks—and three types of links—investments, strategic partnerships, and spin-offs. We represent the nodes as dots and the links as lines in a graphical illustration. The more links one node holds, the bulkier is the respective dot. Light blue dots represent FinTechs, dark blue dots banks, and intermediate blue dots FinTech-banks.

Our initial dataset consists of 436 FinTechs that operate in the German market and which Dorfleitner et al. (2017) identified. We excluded the category Search Engines and Comparison Portals as well as other FinTechs, because these firms might be more similar to comparison portals such as Check24 than start-ups that seek to transform financial services. To create a dataset that mirrors all ties between banks and German FinTechs, we supplemented our FinTech data with information on 62 national and international banks. The majority of banks (84%) and FinTechs (78%) that are active in the Germen market originate from Germany. Foreign companies mostly originate from other European countries, predominantly the United Kingdom, Switzerland, and France.

Four FinTechs also possess a banking license. We subsequently refer to these companies as “FinTech-banks.” We define “FinTech-banks” as start-ups that provide banking services to others and were founded after the year 2002. Given this relatively recent foundation of companies such as solarisBank, biw Bank für Investments und Wertpapiere, and N26 Bank, these banks are more similar to start-ups then traditional German banks such as Deutsche Bank and Commerzbank. A key source of data was the website www.paymentandbanking.com^3^, which continually maps the connections of FinTechs with other companies. The database was previously used in an analysis by Gimpel et al. (2018) to develop a taxonomy of FinTechs start-ups. For 171 FinTechs in our dataset we were able to identify a connection with at least one bank. To categorize the different types of collaborations, we hand-collected additional data from company press releases, annual reports, websites, and trade magazines. We did not identify investments, strategic partnerships, or spin-offs of banks with German FinTechs before 2010 and therefore limited our analyses to the period from 2010 to 2017.

In addition to firm-specific information, such as the founding date and place, we compiled a unique dataset on the professional biographies of 542 FinTech founders, most notably their field of study and former employers. We hand-collected the data from company websites and social media profiles and supplemented them with a survey among the founders. It should be noted that the dataset on German FinTech founders and the dataset on the M&A activities of banks and FinTechs does not perfectly overlap. This is because not all founders could be identified and the dataset on cooperations includes foreign firms as well. For some FinTechs we were not able to find any information about their founders. This is because these FinTechs were often founded by other companies as spin offs (*n* = 35). Spin offs were most often set up in the category crowdfunding (12 spin offs out of 65 crowdfunding FinTechs). Frequently, small firms or non-profit organizations, such as sports clubs or artist associations, founded crowdfunding platforms to raise money related to their specific activities.

### Variables

The *education* of the FinTechs founder is an indicator of the type of innovation a FinTech has developed or aims to develop. In the entrepreneurship literature, the educational background of founders is one of the most widely studied variables. In human capital theory, the variable is often used to understand the transition of individuals to entrepreneurs (Brüderl et al., 1992; Lazear, 2005; Kim et al., 2006). Although we are not aware of any literature that uses the founders' field of study as an indicator of the emergence of a sector and the innovations arising from it, we would expect that founders with a background in science are more prone to science-based innovations while founders with a business administration background have more competences in the implementation of business model innovations.

The *former employer* of a FinTech founder can be considered a proxy for the degree to which the technology must be adapted to a specific context. In the sociology literature and management research on entrepreneurship, the professional background and education are often included under the term “imprinting” (Hambrick and Mason, 1984; Wiersema and Bantel, 1992; Ding, 2011). Here, the assumption is that individuals are subjected to a socialization process during their professional education. This process, in turn, deeply shapes their vision of the firm, values, and information-processing patterns. Research further argues that the professional experience influences founders and their performance because it shapes their networks (Haunschild et al., 1999; Rider, 2012). Moreover, we assume that individuals acquire specific human capital during their professional experience (Becker, 1962; Nonaka, 1994), which can be specific intellectual assets that they later use to found a company. We conjecture that founders who have previously worked for a bank or a management consultancy have specific knowledge about the IT infrastructure of banks and the needs and potential of bank customers. Founders who worked in non-bank-related industries or come from universities are less likely to have such specific knowledge.

The way a company or bank secures *access to a certain technology* also provides insights into the type of technology itself. We define a collaborative tie or alliance as any contractual arrangement to exchange or pool resources between banks and FinTechs or between FinTechs and FinTechs. We differentiate among three types of access to a technology. First, we define investments as the financial interest of one firm in another firm. This category includes the full integration of another company as well as the purchase of shares. The investment of a firm indicates the desire to limit access to a certain technology for rival companies. Second, the establishment of a strategic partnership between firms indicates the non-exclusive access to a technology and suggests that a company wants to participate in the knowledge or the customer base of another company, without taking the risk of a full acquisition. Third, the setup of spin-offs by an already-established company or bank indicates the ability or at least the intention of traditional players to take part in the innovation process.

## Results

### The Origins of FinTech in Germany

The first FinTechs in Germany emerged in the late 2000s. Research has argued that one reason for the emergence of FinTech companies was the recent financial crises (Haddad and Hornuf, 2019). One the one hand, trust in traditional banks was lost, and customers were seeking alternative ways to handle their banking activities. On the other hand, the financial crises made obtaining capital more difficult for firms (Lopez de Silanes et al., 2015), as banks restricted their lending activities. For many bank customers who did not receive capital from traditional banks, crowdlending and crowdfunding platforms provided a much-appreciated alternative. It might further be argued that FinTechs emerged because a bevy of bankers became unemployed due to layoffs at traditional banks. A first hint that the financial crises was indeed a trigger for many FinTech activities is that many FinTech sub-segments started up around the time of the financial crises (e.g., crowdfunding, crowdlending) or in the years that followed (Haddad and Hornuf, 2019).

Moreover, the emergence of many FinTechs around the same time indicates that there was no technological evolution over time, in which one innovation came first and other companies later built on it. A reason for this finding might be that many FinTechs are based on online services and algorithms. As with any software, FinTech innovations are thus a highly context-dependent technology. This means that innovation is not so much driven by scientific findings as by a constant process of adaptation. We explain this observation in detail in the next section.

Furthermore, Dorfleitner et al. (2017) show that the formation of FinTechs is concentrated in specific local areas; more than half of all FinTech formations took place in only four German cities. The uncontested center of the entrepreneurial activity is Berlin, which represents around one-quarter of all FinTech formations in Germany. Berlin is followed by Munich, Frankfurt, and Hamburg. While we also found distinct local centers of the entrepreneurial activity, we could not identify specific intellectual centers from which the formation of FinTechs emanated. In our dataset, we found neither specific universities nor previous employers from which founders of FinTechs originated. The 542 FinTech founders are spread over 169 universities. LMU Munich is the university where most FinTech founders came from (15 founders), followed by the European Business School (11 founders) and the WHU—Otto Beisheim School of Management (10 founders). The former employers of FinTech founders are even more diverse, with founders originating from 268 different employers. By contrast, the majority (92%) of FinTech founders are male, and thus gender is largely homogeneous. The share of female FinTech founders is even smaller than the already low share of women who establish start-ups in the German economy (15%) (Bundesverband Deutsche Startups, 2019), consistent with the historically low participation of women in finance and the science, technology, engineering, and mathematics fields.

Our interpretation of these findings is that the innovation behind FinTechs is not so much science and technology driven as based on learning and doing. This analytical distinction stems from traditional innovation studies that claim that the dynamics of innovation differ by industry (Jensen et al., 2007; Binz and Truffer, 2017). One way to analyze these differences is to determine whether a technology is universal or context dependent. Teece (1998) defines knowledge assets, in contrast with material assets, as assets that, by their nature, cannot readily be sold and bought. We argue, however, that technologies differ in the degree to which they can be exchanged in one interaction. More precisely, technologies can be grouped on a continuum between being universally applicable and highly context dependent. Technologies that tend more toward universality work regardless of their area of application, while more context-dependent technologies must be adapted to specialized conditions (Dasgupta and David, 1994). While in science and technology-driven industries, such as biotechnology, the emergence of intellectual centers such as university or industrial complexes are typical (Powell et al., 2012), the lack of such innovation centers in the financial industry indicates an innovative dynamic that is more strongly driven by factors such as the adaptation to the specific needs of customers.

### Founders of FinTechs: Not Tech Geeks but Businesspeople

**Figure 1:**
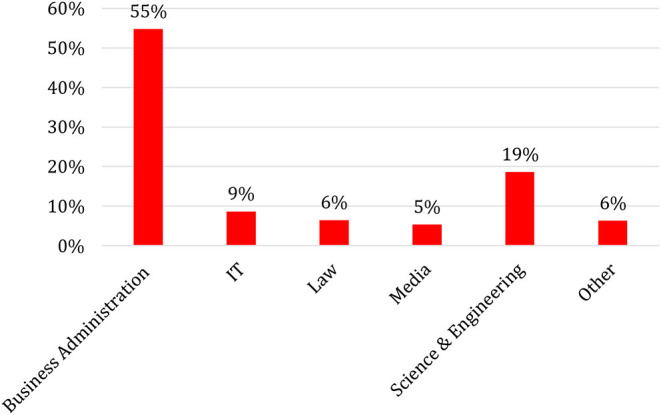
Educational background of FinTech founders (in %), *N* = 422.

FinTechs founders have higher formal degrees than average. Whereas, Metzger (2017) reports that 50% of digital founders in Germany have vocational training as their highest level of education, 92% of the FinTech founders have a degree from higher education institutions. Furthermore, 14% of the FinTech founders hold a doctoral degree, which is far above the average founder education level in Germany. Given that academics generally have better job opportunities, FinTech founders are more likely to be opportunity rather than necessity entrepreneurs. When considering the specific educational background of the FinTech founders, it becomes clear that the overwhelming majority have a business background. Figure 1 shows that 55% of the 348 FinTech founders have a degree in business administration or a related field, such as management, finance, or accounting. Another 19% have a background in science or engineering, and only 9% have a degree in computer science. The remaining 18% have a background in law (6%), media (5%), or other fields (6%). These numbers differ slightly in the various FinTech sub-segments. For example, in the sub-segment of crowdfunding, many founders (14%) have a media background. This can be explained by the specific purpose of *crowdfunding*. In crowdfunding, entrepreneurs intend to raise capital for projects or products from the larger public. This FinTech sub-segment is populated particularly by artists, who develop cultural products that reflect the underlying cultural ideas of their geographic region (Mollick, 2014). In other segments such as *robo advice*, founders more frequently (28%) have a science or IT degree, which is likely due to the challenge to create algorithms that identify an investment strategy; thus, the technological part of the innovation is stronger. In line with human capital theory, our data indicate that the educational background of the FinTech founders relates well to the work content of the respective FinTech sub-segment.

Despite this variation in the sub-segments, the overall trend is clear. Although the digitalization of financial services strengthens the linkage between finance and technology, the entrepreneurial activity is driven by founders from a business background. One interpretation of this empirical finding is that the technological innovation behind FinTechs is rather trivial, while the implementation of innovations, such as the acquisition of customers or the establishment of new standards, is more challenging. This argument is supported by Lerner et al. (2015), who find that financial patents show lower performance in common proxies for the quality of patents, such as the number of citations of scientific publications within the patent or the number of litigations associated with a patent (Lerner, 2002; Tufano, 2003).

**Figure 2:**
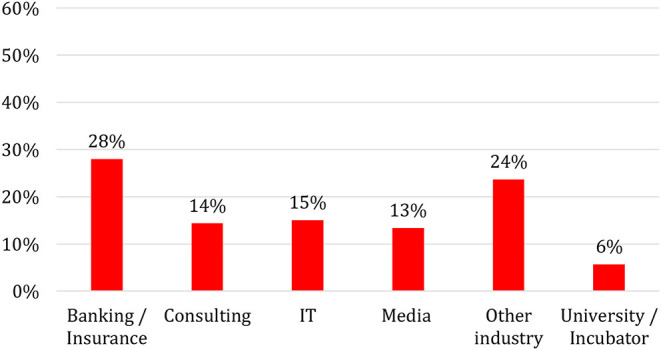
Former employers of FinTech founders (in %), *N* = 450.

An analysis of the former employers of FinTech founders confirms our previous findings. As Figure 2 illustrates, most FinTech founders (28%) previously worked for banks or insurance companies. The share of founders who come from consulting firms is also high (14%). Many management consultants who founded a FinTech company presumably also had a focus on the financial industry. Conversely, we observe a relatively small share of FinTechs that were founded directly out of universities (6%). The number of founders who were previously employed in the IT sector is also rather low (15%), which is surprising given that most FinTech business models are based on software solutions.

We conjecture that the large percentage of FinTech founders with a banking or insurance background is due to the specific requirements of technological innovation in the financial industry. Software in general is a highly context-dependent technology because it requires adaptation to specific contexts. It is virtually impossible to develop software in a context-independent environment and sell a pre-arranged product to customers. Rather, the real work of software developers begins after the first users have begun using a digital product. Programmers of software companies constantly need to fix bugs, adapt their software to a constantly changing hardware environment, and specify their products to the requirements of their customers. The superiority of a software lies not in an initial advantage of a better innovation but in the constant work of contextualization. In the case of banking, the requirement to adapt innovations to a specific context is even stronger, because application programming interfaces (APIs) are not standardized but differ from one bank to another. Often, the nature of an API is only known by the former employees of a specific bank. Although banks are generally willing to share this information, individuals with such specific knowledge and personal connections with bank employees have an advantage.

The necessity to adapt products to existing conditions creates different innovation dynamics than in traditional industries, which are often more science and technology driven. Products that are more science and technology based, such as in the pharmaceutical industry, can be developed in a clean laboratory environment. Although the transformation of these innovations in a real-life context is sometimes more difficult and costlier than expected, the major share of costs in pharma or biotechnology for innovations emerges *ex ante* in the R&D process. In contrast, companies in more context-dependent industries such as software development often must invest more heavily in their service departments after product creation. As we show in the next section, the type of technology also influences the regime of appropriation. While a competitor in a science and technology-driven industry such as biotechnology would have a significant advantage by neglecting the patent of a rival company, someone who illegally uses an algorithm does not automatically have access to the desired product or software, because the software is constantly being improved and developed. In the case of biotechnology, a large share of the technology is expressed in the intellectual property right itself. In the case of software development, a larger share of innovation occurs through the contextualization within a technological architecture.

The contextualization of software and technology is especially important in the financial industry. The current IT infrastructure in the financial industry was created decades ago and evolved incrementally, without a consistent architectural design. Because each bank developed its IT infrastructure to a large extent for itself, contextualization led to a lack of standards (e.g., for APIs). Start-ups therefore cannot create one universal solution for all banks but often must adapt their innovation to the specific technological context of each bank.

### M&A Activities in the Financial Industry: Little Investments, More Strategic Partnerships

**Figure 3:**
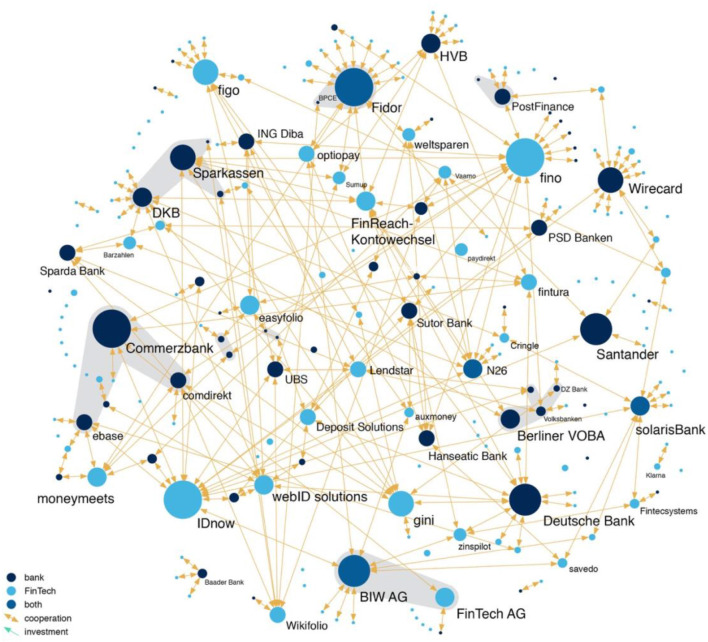
Bank and FinTech cooperation.

**Figure 4:**
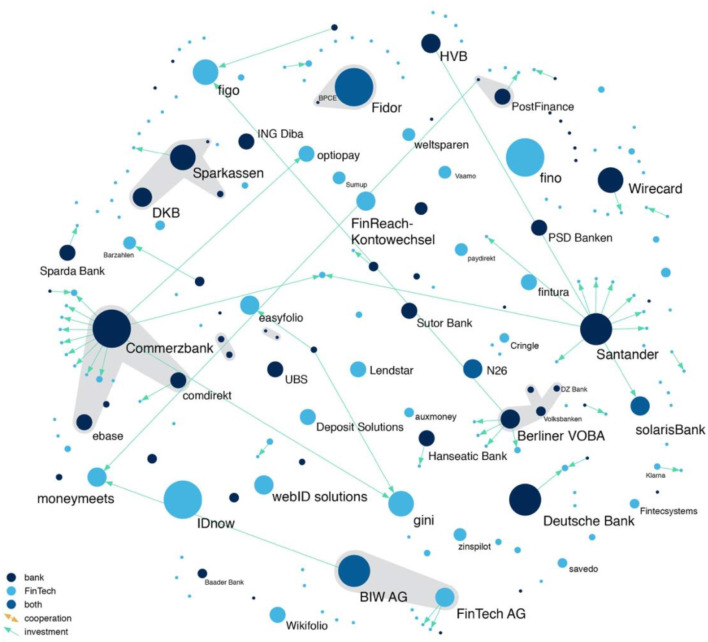
Bank and FinTech investments.

To understand the dynamics unleashed by technological innovation, it is insightful to analyze the incentive structure of companies to integrate or license the new technology. In stark contrast with other emerging industries such as biotechnology, we find evidence that banks do not predominately use the direct integration of start-ups to gain access to the desired technology, but rather employ another form of coordinating intellectual assets: the strategic partnership. Figure 3 maps the contractual links between banks and FinTechs and between FinTechs and FinTechs, while Figure 4 shows only the investment of one company in another. We find that only 19% of all contractual links are actual investments, while the overwhelming majority (74%) are strategic partnerships, and 7% are spin-offs. A strategic partnership is a contractually fixed relationship between two firms. In general, a strategic partnership between a bank and a FinTech or between a FinTech and another FinTech means that one company uses the software of the other company, usually by paying a transaction-based fee. For example, many banks use the video identification tools of FinTech companies, to verify the identity of potential customers in a legally admissible way that is convenient for the customer. In contrast, many FinTechs do not possess a banking license, because their business focus is mainly on front-end operations. For example, many crowdlending portals transfer credit requests to a bank, which consequently originates the loan. Most FinTech-banks such as Fidor Bank, solarisBank, and FinTech AG—biw Bank für Investments und Wertpapiere have realized the potential that stems from the FinTech sector and have specialized in what is called “banking as a service” (BaaS) or “banking as a platform” (BaaP). These banks provide end-to-end processes that ensure the execution of atomic small financial services on demand. BaaP works through APIs provided by the respective FinTech-bank. In other cases, strategic partnerships simply consist of banks making the products of FinTechs available to their customers, such as Consorsbank, which offers crowdinvesting products from Seedmatch and the possibility of social trading through wikifolio. While the bank might only receive a revenue share from the FinTech, the strategic partnership can help the bank maintain customers who might otherwise also switch their core banking activities to a new FinTech competitor. The low percentage of spin-offs (7% of all contractual links) indicates the inability of the established players to actively take part in the innovation process.

To gain a better understanding of the calculus of companies aiming to obtain access to a desired technology, the theories of Teece (1986, 1998) are a useful reference. He shows that the optimal strategy of a company to gain access to a technology depends on the *regime of appropriation*. Referring to Teece's work, Graff et al. (2003) suggest a spectrum of channels to coordinate complementary intellectual assets. The most extreme strategy of a firm is the complete internalization of external assets through integration. On the other end of the continuum stands the purchase of a non-exclusive license for a technology from an independent firm.

The regime of appropriability refers to environmental factors, such as the design of intellectual property rights or the features of the technology itself, that govern an innovator's ability to capture the profits generated by an innovation (Teece, 1986). Moreover, Teece (1986) differentiates between tight and weak regimes of appropriability. A tight regime reflects a situation in which the technology is relatively easy to protect from imitation; correspondingly, a weak appropriability regime describes a situation in which it is almost impossible to protect a new technology from unwanted usage. The characteristics of a regime of appropriability emerge through the interplay between the design of the intellectual property rights (e.g., patents, trademarks, copyrights) and the nature of the knowledge that requires protection.^4^

The context-dependent nature of software affects not only the innovative process *per se* but also the regime of appropriability. We illustrate the interplay of the regime of appropriability and the structure of the industry with another nascent industry with similar starting conditions: early plant biotechnology. In the 1980s, traditional companies secured access to the new technologies of the biotechnology industry by simply acquiring the start-ups. One reason for this drastic strategy was the regime of appropriability. In biotechnology, the weak regime of appropriability impeded the contracting over technologies between different companies and created incentives to fully integrate the start-ups (Graff et al., 2003; Marco and Rausser, 2008; Schneider, 2010). The difficulties in defining intellectual property rights in biotechnology stemmed from the novelty of the field, which led to the paradox situation that the patent offices had to decide on an issue, which was largely unclear even from a scientific perspective. Moreover, in contrast with chemistry or other fields of science, the patent offices could not rely on existing jurisprudence, which makes the results of a court ruling usually more unpredictable. Charles (2002) argues that Monsanto managers realized early on that gens could not be licensed like other technologies such as software. The European counterpart and direct competitor in the early days of agro-biotechnology AgrEvo (now Bayer) did not draw the same conclusions and tried a model of gaining access to various gens and biotechnological tools via license agreements, which did not work out and ended—at least temporarily—in the great defeat of European agrochemical companies in the global seed market (Bijman, 2001; Charles, 2002).

Another observation that differs from adaptation processes in other industries is that FinTech start-ups are active players in reshaping the industry. In other nascent industries (e.g., biotechnology), the start-ups were largely passive, while the dominant players appropriated crucial parts of the new technology (Owen-Smith and Powell, 2003; Powell et al., 2012). In our dataset of German FinTechs, we observe that start-ups themselves take an active role in M&A activities. Some FinTechs began as regular start-ups but eventually received a bank license (e.g., solarisBank) or founded a bank as a spin-off of their FinTech (e.g., FinTech AG—biw Bank für Investments und Wertpapiere). These FinTech-banks coordinated their intellectual assets almost exclusively through BaaS or BaaP. However, we also observe FinTechs that directly integrated other FinTechs by obtaining a minority interest in or acquiring them. In 2013, the Swedish FinTech Klarna, a digital payment service provider, acquired its direct competitor Billpay and, in 2016, Coockies.

The strategies of traditional banks regarding the coordination of their intellectual assets differs widely. While banks such as Santander and Commerzbank largely acquired start-ups from the financial industry, other traditional banks, including HypoVereinsbank and Deutsche Bank, are more cautious about investing in FinTechs. These banks more often engage in strategic partnerships (see Figure 3).

As the emergence of FinTechs is a recent phenomenon, we expect further consolidation in the coming years. However, we argue that the dominance of strategic partnerships as well as the currently missing consolidation is not only a transitional phenomenon that will vanish in the foreseeable future but also has structural reasons that are rooted in the technology and industry itself.

The first reason consolidation of the German FinTech industry is missing lies in the regime of appropriability. In contrast with the biotechnological industry, the appropriability regime of software is tight. This may seem surprising, as the intellectual property protection of algorithms and software is weak in general, especially in Germany and other European countries, where computer programs cannot be patented (Eimer, 2011). Developers of proprietary software must protect their innovations with a weaker intellectual property right—namely, the copyright. In many cases, therefore, the software developers do not depend on intellectual property rights to appropriate their innovations. Software firms often sell software licenses (end-user license agreements) and keep the source code secret. In the case of server-side software, the source code is not protected by a copyright but is kept by a trade secret. Thus, the regime of appropriation is rather tight for software innovations, not only because of the ease of contracting over software but also because explicit knowledge is not an advantage *per se*, as all software products must be adapted to a specific firm and context over time.

Although the license agreements of software companies are not always effective in excluding free-riding customers that do not pay for the service, especially at a business-to-business level, private appropriation of innovations works well in general in the software industry. One reason is that the documentation of software usage takes place automatically through networks and protocols. From the perspective of firms that must contract over technologies, the automatized documentation of usage implies a reduction in transaction costs. While in biotechnology the monitoring and enforcement problems associated with the technology resulted in a structural advantage for large companies that could afford to engage in patent disputes (Haedicke, 2008; Schubert et al., 2011; Gill et al., 2012), the low costs of licensing and monitoring software have led to opportunities for small firms in the financial industry.

A weak regime of appropriability results in a structural advantage for large companies, as is evident in the biotechnological industry. Cost-efficient monitoring and litigating intellectual property right infringements can only be achieved by globally operating companies that maintain branches in different jurisdictions (Haedicke, 2008). The same is true at the business-to-consumer level. The ability to exclude free-riding customers from using the technology often makes a monitoring system necessary (Schubert et al., 2011). The installation and maintenance of a monitoring system is costly, and large companies can more easily realize economies of scale to implement such a system. As a result, a week regime of appropriability increases transaction costs and therefore creates a structural incentive to integrate the company of interest. In contrast, a tight regime of appropriability opens opportunities for small companies (Hall and Zidonis, 2001). Because the contracting over knowledge is reliable, companies can license their technological assets to other firms. In other words, in tight regimes of appropriability transactions costs are lower. Therefore, the incentive for companies to fully integrate a start-up is also lower, as an acquisition is also associated with higher costs and risks.

A second reason that prevents banks from fully integrating FinTechs is the current design of the market infrastructure in the financial industry. The software that underpins the infrastructure was designed for digital requirements that were defined decades ago. The lack of coordination among banks led to siloed data stores maintained by individual participants. Most experts agree that players in the financial industry must reconcile the current system sooner or later, especially regarding the status of transactional data. The current lack of coordination in common technological standards and banking functions leads to a hesitation among banks to fully integrate FinTechs. The coordination of intellectual assets of strategic partnerships, however, enables banks to appropriate the knowledge of FinTechs without needing to make fundamental decisions about the future of their IT infrastructure. Finally, strategic partnerships are an efficient way for banks to overcome their cultural legacy, extensive regulatory provisions, and compliance issues, which also allows them to approach different technologies without having to commit to a specific one.

## Conclusion

We began this article by stating that the digitalization of financial services could potentially turn the industry upside down. Not only do FinTechs have a streamlined cost structure, but they also provide novel services to customers, such as fully digital financing or investment solutions. In contrast, most of the traditional banks rely on a historic and expensive IT infrastructure. Our analysis indicates that the current wave of digitalization does not unleash the same groundbreaking dynamics as other innovations such as biotechnology. We explain that the hesitation of many banks to fully endorse the new possibilities of digitalized financial services is due to the context dependency of the software and the tight appropriation regime. In other words, the characteristics of the technology allow banks to participate only partly in the new wave of digitalization through strategic partnerships, without needing to change their own outdated IT infrastructure.

Our results might be only one part of the story. Our analysis is restricted to the German market, and different regions of the world might show another pattern. Moreover, we only focus on a specific type of FinTech. Although the current FinTechs challenge the traditional banks through their digitally optimized cost structure and their affinity to new technology, they are not a self-sustaining alternative to the banking system. Almost all currently active FinTechs rely on banks, mostly because they do not possess a banking license, which is required to conduct core banking operations (taking deposits and extending loans).

In the future, another type of FinTech might become more important. FinTechs do not just offer services that work in parallel to the current system but also provide technologies and services that aim to fully replace the current structures and organizations of the financial industry. These FinTechs either possess a banking license or rely on self-sufficient systems such as blockchain, the technology that stands behind Bitcoin and other crypto currencies, and thus could supersede traditional banks (Tapscott and Tapscott, 2016). Blockchain is an open, distributed ledger that can verifiably and permanently record transactions between two parties. As contracts and records of transactions are the defining institutions of the economy, the technological transformation of these institutions has the potential to cause a deeper social change process than the current FinTechs have. Blockchain start-ups offer technological solutions for various problems. The first product based on blockchain, Bitcoin, for example, promises to be a currency that is protected from the access of central banks during economic crises (Valkenburgh, 2016). Digital Asset Holdings, a software firm located in New York City, offers software solutions based on the distributed ledgers technology for the entire architecture of the financial industry, such as central clearing houses and central securities depositories (Digital Asset, 2016). Thus, unlike all other FinTech innovations, blockchain has the potential to replace the financial industry as such. For this to happen, the new technology needs to be trusted by all market participants. Trust, however, is a critical asset that is built up only in the long run.

Because the financial industry was until now not capable of agreeing on common standards for a new IT infrastructure and because of the political inability to solve this coordination problem, the strategic development of firms and the future of the blockchain technology as such are highly unclear. Iansiti and Lakhani (2017) compare the current situation of blockchain to the early days of e-mail. Before the adoption of the transmission control protocol and Internet protocol (TCP/IP), the telecommunications architecture was based on *circuit switching*, a method in which the connection between two parties had to be physically pre-established with an electrical circuit. The adoption of TCP/IP not only created a new communication architecture but also paved the way for entirely new technological applications such as the World Wide Web.

Technologies that aim to replace the current ones create an enormous coordinative challenge for the economic agents involved, as success depends entirely on the establishment of novel, effective institutions, and organizations. This challenge can be met by a company, an industrial pressure group, or the state by implementing legal regulations. Many studies in the field of comparative political economy have shown that different types of economies have established varying ways of dealing with coordination problems in relation to technology and platform services. Hall and Soskice (2001) argue that in liberal economies (e.g., United States), firms compete over standard settings, while in coordinated economies (e.g., Germany), industry-specific networks coordinate a collaborative process of standard settings. To enable complex technological adaptation, in some industries the state must step in, if firms or other organizations fail to coordinate on technological standards (Casper and Soskice, 2004).

The infrastructure that underpins the financial industry was developed decades ago. Although it incrementally evolved, the current system is largely unable to meet the current regulatory and market needs of the financial system. It is an open secret that international banks such as Goldman Sachs, J.P. Morgen, and Citigroup as well as companies such as Deutsche Börse and PricewaterhouseCoopers are actively acquiring blockchain start-ups and applying for patents on parts of the blockchain technology on their own business models. The makeover of the digital architecture of financial markets, the reconciliation of the siloed data stores, and the agreement on standards is an enormous coordinative challenge. It is still an open question, however, whether the financial industry will be able to deal with this challenge. The danger of leaving this challenge to market players is that banks either are not capable of agreeing on a common standard or, even worse, will decide on the wrong standard. The task of designing a novel IT architecture for the financial markets is therefore also a political question, which can only be answered democratically.

## Data Availability Statement

The datasets generated for this study will not be made publicly available. The data was partly obtained from https://paymentandbanking.com. Publishing the curricular information of fintech founders might violate their privacy.

## Author Contributions

Both authors have made substantial, direct, and intellectual contribution to the work, and approved it for publication. Both authors were involved in data collection and theoretical framing. Data analysis was conducted by BB.

### Conflict of Interest

The authors declare that the research was conducted in the absence of any commercial or financial relationships that could be construed as a potential conflict of interest.

## Footnotes

^1^We apply the typology that was developed by Dorfleitner et al. (2017), which focuses on the German FinTech market. Another typology of FinTech business models was suggested by Eickhoff et al. (2017).

^2^See https://www.paydirekt.de/ueberuns/.

^3^http://www.paymentandbanking.com kindly provided their permission for the usage of this data.

^4^The knowledge economics and organizational sociology literature streams describe several classes of problems related to the tasks of coordinating knowledge between different units or companies and determining whether an appropriability regime is tight or weak (Pisano, 1990; Nonaka, 1994; Heller and Eisenberg, 1998; Graff et al., 2003; Brandl and Glenna, 2016): (1) monitoring and enforcement problems due to the difficulty in determining who is using a certain technology and whether the users pay for it, (2) entitlement problems due to poorly defined and/or silent blocking of intellectual property rights, and (3) monitoring problems due to the difficulty of measuring and therefore contracting over certain types of technology or knowledge.
